# Maggot magic: Unveiling the superpowers of larval therapy in malignant wound management

**DOI:** 10.1016/j.jdcr.2024.09.012

**Published:** 2024-09-30

**Authors:** Verena Isak, Joseph Stoll, Cara Norelli, Alina Markova

**Affiliations:** a Department of Dermatology, Weill Cornell Medicine, New York, New York; b Department of Dermatology, Memorial Sloan Kettering Cancer Center, New York, New York

**Keywords:** debridement, Kaposi sarcoma, larval therapy, malignant wound, wound care

## Introduction

Chronic wounds affect about 2% of the US population, with a prevalence of 3% in those aged ≥65 years.^1^ Malignant wounds represent a subtype of chronic wounds that is seen in about 5% to 14% of patients with advanced stages of cancer.^2^^,^^3^ Infiltration of malignant cells into the skin, either via metastasis or direct extension from an underlying tumor, lead to oftentimes large, fungating wounds. The unique environment that is created through the rapid growth, neovascularization and necrosis seen in tumors comes with several challenges, both on a physical and emotional level, and with management of these wounds. Malignant wounds offer an ideal breeding ground for numerous pathogens and ongoing colonization.^4^ Symptoms including pain, pruritus, exudate, malodor, bleeding, and the constant visible reminder of malignancy, lead to significant emotional distress of patients and their relatives, and ultimately significantly deteriorate a patient’s quality of life.^5^

Management of malignant wounds is very challenging, with often limited success. Given the underlying etiology of a growing malignancy, healing is often not the primary goal in the management of malignant wounds, but rather the management of symptoms including pain, pruritus, malodor, and discharge, with the goal of improving the patient’s quality of life.^5^ Absorbent dressings, topical and oral antibiotic, and analgesic therapies are the mainstay in managing malignant wounds.

An accidental infestation of a wound by maggots is a rare complication in patients with chronic wounds. In a controlled setting, medical maggot therapy can be used as a natural way of debriding necrotic tissue. Several studies have compared maggot debridement therapy with conventional treatment in pressure, diabetic, venous, or mixed ulcers; results suggest maggot therapy to be equal or faster for debridement of necrotic tissue, promotion of granulation tissue growth and reduction of wound surface area.^6^^,^^7^ Although malignant wounds were not included in aforementioned studies, and only 1 case report of successful debridement via maggot therapy of a malignant wound that failed other attempts of debridement, can be found in the literature,^8^ the fast and effective debridement of nonviable tissue performed by maggots offers an alternative to other debridement methods in especially fungating malignant wounds with a high percentage of nonviable tissue.

## Case

**Fig 1:**
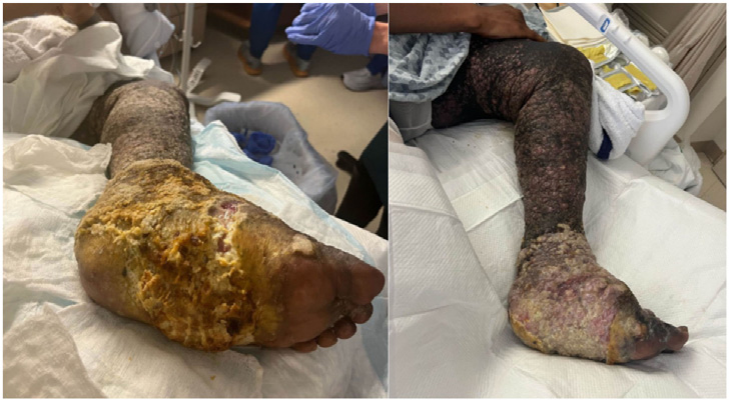
Left lower extremity with verrucous hyperkeratotic plaques involving the plantar and dorsal surface of the left foot, and with significant debridement of hyperkeratotic plaques and visualization of pink granulation tissue status post larval therapy.

**Fig 2:**
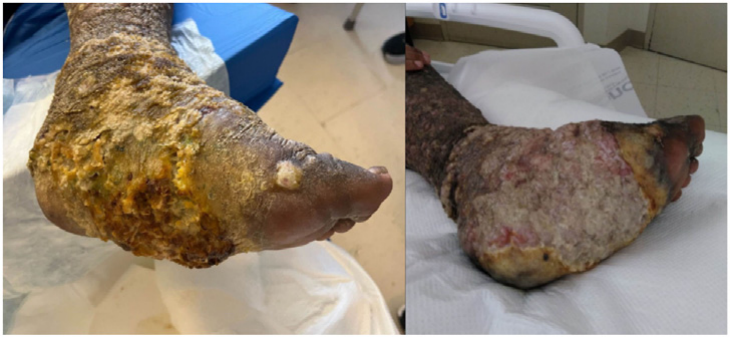
Close-up photo of the left foot with hyperkeratotic verrucous, fungating plaques on the left plantar and dorsal foot, and pink granulation tissue status post debridement via larval therapy.

A 39-year-old man with a history of HIV/AIDS and Kaposi sarcoma with progression of disease despite multiple lines of treatment (paclitaxel, doxorubicin, pembrolizumab, pomalidomide, gemcitabine, and radiation therapy to left foot) presented for management of left side of the lower extremity malignant wound and increased swelling of his left extremity. Multiple polymicrobial superinfections over the past few months required several courses of broad-spectrum oral and intravenous antibiotics. On examination, the left leg showed striking edematous enlargement and extensive hyperkeratotic brown plaques with cobblestone and papillated appearance (Fig 1, *left*). On the left foot, there was a fissured ulcer with purulent discharge and foul odor on the medial aspect, surrounded by hyperkeratotic fungating plaques (Fig 2, *left*). Imaging (computed tomography and magnetic resonance imaging) showed no signs of osteomyelitis. The superinfection was treated with broad-spectrum antibiotics. General surgery was consulted to evaluate for debridement, however recommended conservative wound care because of extensive underlying disease, recent radiation therapy, and concern for poor wound healing. Given a history of accidental maggot infestation that brought the patient some relief from the pain and heaviness of the fungating tumor, he inquired about medical maggot therapy. Medical maggots were ordered and shipped overnight from Monarch Labs, LLC. Five hundred and eight larvae of *Lucilia sericata* spp. in moist gauze were placed on the wound on the left foot covered by a polyester net dressing sock over the wound and the dressing was secured per the manufacturer’s instructions with a hydrocolloid dressing with adhesive glue and transparent film dressing. A thin layer of gauze was loosely wrapped around the leg to allow the larvae to breathe and was changed daily as needed for soiling. After incubation for almost 48 hours, the dressing was removed, and the medical maggots, and loose, necrotic tissue, were removed using irrigation with sterile saline, wet gauze, a scalpel, and forceps; the results were significant debridement of necrotic tissue with a now visualized pink wound bed (Figs 1 and 2, *right*), and a subjective improvement in the perceived heaviness of the foot by the patient.

## Discussion

Although the possible employment of maggots in debridement of necrotic wounds has been known for centuries and historic reports of its positive effects on wound healing have been found from Native American, Mayan, and Aboriginal tribes and reports stemming from soldiers in the American Civil War, World War I and II, maggot therapy has fallen into oblivion with the emergence of antiseptic and antibiotic therapies in medicine. The Food and Drug Administration has approved medical maggot therapy as a form of biotherapy for the use in chronic wounds in humans in 2004.^9^

Medical maggot therapy is an inexpensive and natural way of debriding large amounts of nonviable tissue in a short period of time, allowing for the wound bed to be exposed and to subsequently granulate.

Larvae debride wounds by dissolving necrotic and infected tissue through proteolysis via digestive enzymes, secrete antimicrobial molecules, and stimulate growth of granulation tissue.^10^ Because maggots only eat dead or infected tissue, but not healthy tissue, they offer an alternative in otherwise challenging wounds such as the fungating and ulcerated wound in our patient, in which the primary goal is symptom management and reduction of the risk of superinfections.

Limitations of medical maggot therapy include the selection of the right wound, which is pertinent to allow for larvae to survive and thrive: moist, exudative wounds with easy oxygen access are ideal for medical maggot therapy; additionally, wounds must not have any connection to body cavities.

Given the progressive growth that can often be observed with malignant wounds, a natural debridement option such as medical maggot therapy offers an excellent addition to palliative wound care and may help reduce both the emotional and physical burden that fungating malignant wounds can create for patients and their family members. As such, wider use in the management of malignant wounds should be further explored in the appropriate setting and patient.

## Conflicts of interest

Dr Markova has received research funding from Amryt Pharma, 10.13039/100017655Incyte Corporation, Kintara Therapeutics, 10.13039/100004336Novartis, and Novocure; is a consultant for ADC Therapeutics, Alira Health, AstraZeneca, Blueprint Medicines, 10.13039/100020154Protagonist Therapeutics, OnQuality, Sanofi-Genzyme, and Janssen; and receives royalties from UpToDate. Drs Isak and Stoll and Author Norelli have no conflicts of interest to declare.
